# *Cell Communication and Signaling *is becoming the official journal of the Signal Transduction Society

**DOI:** 10.1186/1478-811X-6-1

**Published:** 2008-08-06

**Authors:** Stephan M Feller, Ralf Hass, Ottmar Janssen, Karlheinz Friedrich

**Affiliations:** 1 The Weatherall Institute of Molecular Medicine, University of Oxford, London, UK; 2 Signal Transduction Society, Germany; 3 Hannover Medical School (MHH), Hannover, Germany; 4 Institute of Immunology, University of Kiel, Kiel, Germany; 5 Institute of Biochemistry, University of Jena, Jena, Germany

## Editorial

The Signal Transduction Society (STS) [1] is delighted to join BioMed Central with an open access journal. Over the last years, our society members have increasingly appreciated that access to scientific information generated by publicly funded academic research must not be restricted by commercial interests. With overwhelming support of the society members, the presidial council and advisory board of the STS are therefore now taking action and moving from an access-restricted print journal to online open access publishing.

We are convinced that unduly limiting the flow of scientific knowledge has a negative impact on the development of benefits for mankind and believe that BioMed Central is a highly valuable platform that is vital not only to scientists but to society in general and hence deserves our strong support.

The need for free information flow within the academic community is especially true for rapidly evolving fields such as cell signaling, which is now entering a new and exciting era of development. The foundations in our molecular understanding, laid by numerous colleagues over the last decades, are now beginning to bear fruit in the form of an increasing number of targeted, signal transduction-modulating drugs entering clinical use. Over a dozen examples can be found in current cancer therapies alone, with more than a hundred further anti-cancer drug candidates currently undergoing clinical evaluation. Nevertheless, much still needs to be done to improve the outcomes for countless severely suffering patients.

*Cell Communication and Signaling *(*CCS*) and the STS are committed to playing an active part in the signal transduction research community by providing a stimulating and collegial forum for the speedy publication of state-of-the art research and discussions.

*CCS *intends to cover all aspects of cell signaling in health and disease, from ultrastructural and molecular studies with atomic resolution to signaling pathways, molecular machines and signaling networks and further on to various forms of inter-organismal signaling as, for example, exerted by bacterial and viral pathogens. We will accept timely reviews and commentaries as well as original articles with high-quality data in several formats (further details below). Supported by the journal's editorial board members and solicited expert reviewers, the editors will make it a priority to ensure that publications are not unduly delayed. It has become an aggravating fact of life for many scientists that even well-prepared manuscripts are rarely accepted without requests to perform multiple additional experiments. Some manuscripts undergo four or five rounds of resubmission, often resulting in publication delays for several years. This does not go down well with many funding bodies and puts especially young investigators at a disadvantage. The friendly reviewers' comment "This is a great paper well worth publishing as soon as possible provided the authors can submit further supporting data from a mouse knock-in and a genome-wide gene expression array analysis of no less than 500 patient samples" has become a standing joke at many scientific gatherings. In far too many cases, delaying manuscripts has less to do with improving manuscript quality and more with conflicting interests of various kinds.

*CCS *will not be a part of this. We recognise that science is a continuous process that is never finished and that real proof comes primarily from independent confirmations by other researchers. This is often not possible unless a study, albeit incomplete, is publicly accessible.

If an original manuscript's data are novel, important and technically sound, if the conclusions drawn are not severely flawed, the quality of results and presentation are state-of-the-art and the ethics acceptable, the work will be published. The editors do, however, reserve the right to add an editorial comment if they feel that this is important for the scientific community. Authors may be invited to comment on these. Reviewers will be requested to provide fair and constructive criticism that helps the authors to improve their manuscripts.

We intend to publish the following types of contributions: original research, reviews, commentaries, debate articles, hypotheses, methodology articles and short reports. Further details regarding these types of article is available via the *Cell Communication and Signaling *about page [2].

As many readers will already know, it is not possible to make high-quality open access publishing a reality without a cost contribution of the authors. As such, *Cell Communication and Signaling *levies an article-processing charge (APC) for each accepted manuscript. There are currently over 300 institutions with BioMed Central membership [3] whereby the APC will be fully or partially covered by the membership, and a number of funding bodies allow their grants to be used to cover the APC [4]. Authors can request a waiver of the APC through the submission system and these will be considered where there is a genuine inability to pay.

With the support of the STS, of our editorial board members and of our valued colleagues around the globe, we believe that *CCS *will achieve its goal, which is to become an appreciated place to quickly disseminate exciting findings from and to the signaling community and beyond. We are looking forward to working with you all.

With warm regards,

Stephan M. Feller, Editor

Ralf Hass, President STS & Associate Editor

Ottmar Janssen, Presidial Council STS & Associate Editor

Karlheinz Friedrich, Presidial Council STS

## Competing interests

The authors declare that they have no competing interests.
